# An Algorithm for Salvage of Implant‐Threatening Complications in Breast Reconstruction

**DOI:** 10.1155/tbj/4668584

**Published:** 2026-08-27

**Authors:** Elizaveta Kouniavski, Shani Gilron, Moran Lemberger, Pnina Ciobotaro, Dana Egozi, Sharon L. Kracoff-Sella

**Affiliations:** ^1^ The Department of Plastic and Reconstructive Surgery, Kaplan Medical Center, Affiliated to the Faculty of Medicine, The Hebrew University of Jerusalem, Rehovot, Israel, huji.ac.il; ^2^ The Faculty of Medicine, The University of Tel-Aviv, Chaim Levanon St 55, Tel Aviv-Yafo 6997801, Israel

**Keywords:** breast reconstruction, direct-to-implant, immediate breast reconstruction, implant-based reconstruction, postoperative complications, surgical site infection

## Abstract

**Background:**

Implant‐based breast reconstruction is associated with postoperative infectious and wound‐related complications that may compromise reconstructive outcomes, particularly in cohorts predominantly undergoing immediate reconstruction. Optimal management remains controversial, with no universally accepted salvage algorithm.

**Methods:**

We conducted a retrospective cohort study of 198 patients (285 breasts) who underwent implant‐based reconstruction between 2013 and 2023, the majority of whom underwent immediate reconstruction. Implant‐threatening complications were defined as surgical site infection according to Centers for Disease Control and Prevention (CDC) criteria, full‐thickness skin necrosis, or deep wound dehiscence. A standardized institutional salvage protocol incorporating antibiotic therapy with or without surgical intervention was applied. Outcomes included implant salvage and final reconstruction status at the end of follow‐up, and logistic regression was used to identify predictors of salvage failure.

**Results:**

Implant‐threatening complications occurred in 28.3% of patients, with salvage achieved in 51.8% of cases. All patients in the salvage group achieved final reconstruction, compared to 44.4% in the nonsalvage group (*p* < 0.01). The most common complications were skin necrosis and wound dehiscence, followed by infection, and Gram‐negative organisms predominated among culture‐positive cases. Resistant organisms (*p* = 0.04), implant extrusion (*p* = 0.008), and prior radiation therapy (*p* = 0.019) were independent predictors of salvage failure.

**Conclusion:**

A protocol‐based salvage approach may facilitate implant preservation and improve final reconstruction rates. Early, case‐specific conservative management, tailored to local microbiologic patterns, may optimize outcomes.

## 1. Introduction

Infectious and wound‐related complications following mastectomy and implant‐based breast reconstruction remain a significant clinical challenge, contributing to patient morbidity, prolonged hospitalization, and increased healthcare costs [1]. These complications may compromise reconstructive outcomes and, if not managed appropriately, can lead to early implant failure. In addition to their immediate impact, such events are associated with a higher likelihood of revisional procedures and account for a substantial proportion of secondary operations in breast reconstruction [2, 3]. Traditionally, severe periprosthetic infections have been managed with implant removal followed by delayed reconstruction [4, 5]. While this approach may effectively control infection, it carries important drawbacks, including soft tissue contraction, distortion of the breast envelope, and a reduced likelihood of completing reconstruction. Moreover, delayed reconstruction imposes additional physical and psychological burden on patients and often entails more complex surgical interventions. In recent years, there has been growing interest in salvage strategies aimed at preserving the implant and maintaining the reconstructive course. These approaches range from antibiotic therapy alone to combined medical and surgical interventions, including surgical debridement, implant pocket irrigation through indwelling drains or catheter‐based systems, and adjunctive measures such as antibiotic‐impregnated beads or negative pressure wound therapy. However, reported success rates vary widely, and there is currently no universally accepted management algorithm. This variability reflects differences in patient populations, reconstructive techniques, definitions of infection, and local microbiologic profiles, as well as the increasing prevalence of resistant bacterial strains [6]. An additional challenge lies in the heterogeneity of clinical presentation, as not all implant‐threatening complications present with overt infection. Conditions such as wound dehiscence, skin flap necrosis, and implant exposure may precede or coexist with infection and similarly jeopardize reconstructive success. Early identification and timely intervention in these cases may play a critical role in preventing implant loss. In this context, we present our institution’s protocol‐based approach for the management of implants at risk following mastectomy and implant‐based reconstruction. This strategy incorporates early recognition, targeted antibiotic therapy, and selective surgical intervention with the goal of preserving the original implant whenever feasible. The primary aim of this study was to evaluate the efficacy of this salvage approach in avoiding implant removal. The secondary aim was to assess whether successful implant salvage is associated with higher rates of final reconstruction at the end of follow‐up, compared to explantation and delayed reconstructive attempts.

## 2. Methods

### 2.1. Study Design and Population

We conducted a retrospective cohort study of patients who underwent implant‐based breast reconstruction at our institution between 2013 and 2023. Data were collected from electronic medical records and included patient demographics, comorbidities, oncologic and reconstructive details, and postoperative complications.

This study was approved by the Institutional Review Board of Kaplan Medical Center (approval number: 46‐16), and the requirement for informed consent was waived due to the retrospective design. Inclusion criteria comprised women aged ≥ 18 years who underwent immediate or delayed implant‐based reconstruction following mastectomy. Patients undergoing contralateral augmentation for symmetry were also included.

### 2.2. Definitions and Outcomes

Infectious complications were defined and classified according to the most recent Centers for Disease Control and Prevention (CDC) criteria [7], independent of the treatment administered at the time of presentation. Infections were categorized as superficial or deep.

Patients were classified as having an “implant at risk” if they developed infection meeting CDC criteria, full‐thickness skin necrosis, deep wound dehiscence with actual or impending implant exposure, or a seroma/hematoma associated with clinical signs of impending implant compromise that did not fulfill CDC criteria for surgical site infection. Cases of isolated cellulitis were excluded from these definitions. The primary outcome was successful implant salvage, defined as retention of the original implant without the need for explantation. The secondary outcome was final reconstruction, defined as the presence of a reconstructed breast at the end of follow‐up, regardless of reconstructive modality. Complications were analyzed on both a per‐patient and per‐breast basis. Although some patients experienced multiple complications, each patient was classified according to the primary presenting complication. Timing of complications was categorized as early (≤ 30 days), late (31–90 days), and very late (> 90 days) [8].

### 2.3. Surgical Technique and Perioperative Management

The majority of patients underwent immediate, single‐stage direct‐to‐implant (DTI) reconstruction using acellular dermal matrix (ADM). A smaller subset underwent two‐stage reconstruction with tissue expanders. Most implants were initially placed in a submuscular plane, with a transition toward prepectoral reconstruction adopted in later years. Standard perioperative antibiotic prophylaxis and drain management protocols were applied.

### 2.4. Salvage Protocol

**FIGURE 1:**
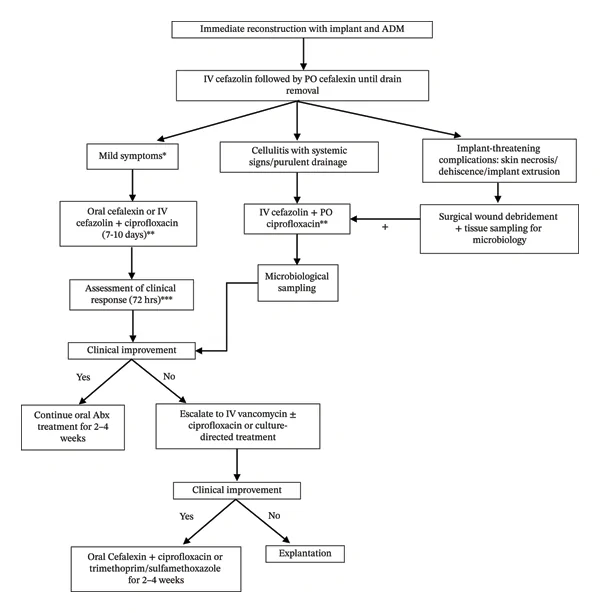
Institutional protocol for management of implant‐threatening complications following implant‐based breast reconstruction. This algorithm outlines a stepwise approach based on clinical presentation, including mild local symptoms, suspected infection with systemic features, and implant‐threatening complications such as necrosis, wound dehiscence, or implant exposure. Management includes empiric antibiotic therapy, microbiological sampling, and early surgical intervention when indicated. Clinical response is reassessed at defined intervals, and treatment is escalated in cases of inadequate improvement. Implant salvage is pursued whenever feasible, while failure of conservative or surgical management leads to explantation and consideration of delayed reconstruction. ^∗^Mild symptoms are defined as localized erythema and/or warmth without systemic signs. ^∗∗^Initial empiric salvage treatment—high dose cefalexin (1 gr TID), ciprofloxacin (750 mg BID), and cefazolin (2 gr TID). ^∗∗∗^Clinical response—improvement in local signs and/or systemic parameters within 72 h. PO: per os, IV: intravenous, ABX: antibiotics, ADM: acellular dermal matrix.

A standardized institutional protocol was applied for the management of implants at risk. Treatment strategies were guided by clinical severity and included antibiotic therapy, image‐guided drainage when indicated, and selective surgical intervention such as debridement and wound closure. Escalation of care was based on clinical response and microbiologic findings. Explantation was reserved for cases failing conservative or surgical salvage efforts. A detailed schematic of the protocol is provided in Figure 1.

### 2.5. Microbiologic Assessment

Cultures were taken in any implant‐threatening complication with available material for sampling. This included debrided tissue in cases of necrosis or dehiscence, aspirated seromas, or blood cultures in cases with systemic fever. Antibiotic therapy was subsequently tailored according to culture results and sensitivities, in collaboration with infectious disease specialists when required.

### 2.6. Statistical Analysis

Categorical variables are presented as frequencies and percentages, and continuous variables as means with standard deviation or medians with range, as appropriate. Group comparisons were performed using Pearson’s chi‐square test. Logistic regression analysis was used to identify independent predictors of salvage failure, with model fit assessed using the Hosmer–Lemeshow test. Statistical significance was defined as *p* < 0.05. Data were analyzed using IBM SPSS Statistics for Windows, Version 27.0 (IBM Corp., 2020).

## 3. Results

### 3.1. Patient Characteristics and Reconstruction Details

A total of 198 patients (285 breasts) who underwent implant‐based breast reconstruction between 2013 and 2023 were included. The majority of patients (82%, 162/198) underwent DTI reconstruction. The median follow‐up time was 386 days (6–3122); only 6 patients had less than 30 days follow‐up. Baseline cohort characteristics are presented in Table 1.

**TABLE 1:** Patient characteristics, risk factors, and surgical procedure performed.

Characteristic	*n* (%) or value
Age at surgery (years)	50.1 (28–75)^†^
Follow‐up duration (days)	386 (6–3122)^†^
Surgical approach	
➢ Skin‐sparing mastectomy (SSM)	103 (52.0)
➢ Nipple‐sparing mastectomy (NSM)	81 (40.9)
➢ Simple/radical mastectomy	3 (1.5)
➢ Other^∗^	11 (5.6)
Implant position	
➢ Submuscular	155 (80.7)
➢ Pre‐pectoral	37 (19.3)
Risk‐reducing surgery	25 (12.6)
Reconstruction type	
One‐stage (direct‐to‐implant)	162 (81.8)
Two‐stage (tissue expander)	36 (18.2)
Prior surgery on same breast	36 (18.2)
Comorbidities	
➢ Smoking	32 (16.3)
➢ Diabetes mellitus	19 (9.6)
Prior treatments	
➢ Prior radiation therapy	29 (14.6)
➢ Neoadjuvant chemotherapy	40 (20.2)

*Note:* Patient demographics, clinical characteristics, and surgical details of the study cohort. Values are presented as number (percentage) unless otherwise specified. Surgical approach, implant position, reconstruction type, comorbidities, and prior oncologic treatments are summarized for the overall cohort.

^†^Values are presented as median (range).

^∗^Other includes oncoplastic reconstruction with implants, implant exchange, or augmentation ± mastopexy for symmetry.

### 3.2. Complications

Complications occurred in 56 patients (28.3%), involving 65 breasts (22.8%). The most common complications were skin flap necrosis and wound dehiscence, followed by infection and seroma/hematoma. Multiple complications frequently co‐occurred. Most complications developed within 30 days postoperatively (41/56, 73.2%), while 11 cases (19.6%) occurred between 31 and 90 days, and 4 cases (7.1%) presented after 90 days. Clinical presentation of infectious cases is detailed in Table 2.

**TABLE 2:** Postoperative complications.

Complication	*n* (%)^†^
Any complication	56 (28.3)
Type of complication	
➢ Skin necrosis	18/56 (32.1)
➢ Spontaneous wound dehiscence	16/56 (28.6)
➢ Seroma/Hematoma	7/56 (12.5)
➢ Infection (overall)	15/56 (26.8)
⁃ Deep tissue infection^∗^	7/56 (12.5)
⁃ Purulent discharge	8/56 (14.3)

*Note:* Postoperative complications among patients undergoing implant‐based breast reconstruction. Values are presented as number (percentage). Percentages for individual complications are calculated among patients with complications. Some patients experienced more than one complication but were classified according to the primary presenting complication.

^†^Percentages are calculated among patients with complications (*n* = 56).

^∗^Defined according to CDC criteria.

### 3.3. Salvage and Reconstruction Outcomes

Among patients with implant‐threatening complications, 30 patients (37 breasts) underwent surgical debridement as part of a salvage attempt. Overall, 29 of 56 patients (51.8%) were successfully salvaged, while 27 patients (48.2%) required implant explantation‐7 immediately and 20 following failed salvage attempts.

All patients in the salvage group (29/29, 100%) achieved final reconstruction at the end of follow‐up, compared to 12/27 patients (44.4%) in the explantation group (*p* < 0.01). Overall, final reconstruction was achieved in 73.2% (41/56) of patients with implants at risk.

### 3.4. Microbiological Findings

**FIGURE 2:**
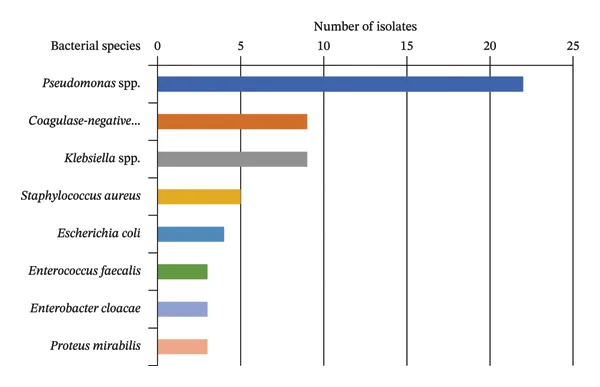
Bacterial species isolated from positive implant cultures. Microbiological data were available for 63 cases, of which 40 yielded positive cultures. The most frequently isolated organisms were *Pseudomonas* spp., coagulase‐negative *Staphylococci*, and *Klebsiella* spp., followed by *Staphylococcus aureus*, *Escherichia coli*, *Enterococcus faecalis*, *Enterobacter cloacae*, and *Proteus mirabilis*.

Microbiological data were available for 63 cases, of which 40 (63.5%) yielded positive cultures. Among the 40 positive cultures, 25 (62.5%) grew Gram‐negative bacteria, 10 (25.0%) grew Gram‐positive bacteria, and 5 (12.5%) yielded mixed or atypical organisms (Figure 2). The most commonly isolated organisms were *Pseudomonas* species (55%), followed by coagulase‐negative *Staphylococci* and *Klebsiella* species (22.5% each). Resistant strains were identified in 13 of 40 cases (32.5%).

### 3.5. Predictors of Infection and Salvage Outcomes

The presence of seroma or hematoma was associated with infectious complications (*p* = 0.048). Multivariable logistic regression demonstrated that resistant organisms (OR 0.078, 95% CI = 0.007–0.894, *p* = 0.04), implant extrusion (OR 0.04, 95% CI = 0.004–0.437, *p* = 0.008), and prior radiation therapy (OR 0.027, 95% CI = 0.001–0.547, *p* = 0.019) were independently associated with reduced odds of successful implant salvage. No significant associations were observed between successful implant salvage and implant position, diabetes, chemotherapy, or smoking status.

### 3.6. Factors Associated With Final Reconstruction

In multivariable analysis, no evaluated variables, including reconstruction type, smoking status, prior radiation, seroma/hematoma, or implant extrusion were significantly associated with final reconstruction outcomes (all *p* > 0.05).

### 3.7. Late Complications

Capsular contracture was documented in 34 patients (23.8% of 143 patients with available data). Documentation was incomplete in a proportion of cases, as this outcome was not predefined.

## 4. Discussion

In this study, we evaluated a protocol‐based approach for managing implant‐threatening complications following implant‐based breast reconstruction. Complications occurred in 28.3% of patients, most commonly skin necrosis and wound dehiscence. Using our institutional protocol, implant salvage was achieved in 51.8% of cases. Notably, all patients in the salvage group ultimately achieved final reconstruction, compared to less than half of those undergoing explantation. In addition, resistant organisms, implant extrusion, and prior radiation therapy were identified as independent predictors of salvage failure.

Infectious complications in implant‐based breast reconstruction remain challenging to define and manage, with reported rates varying widely across studies [6, 9]. This variability is partly attributable to inconsistent definitions and reporting standards. In our study, infections were classified according to the most recent CDC criteria [7], allowing for a standardized and reproducible assessment independent of treatment decisions at presentation.

Our microbiological findings demonstrated a predominance of Gram‐negative organisms, in contrast to the traditionally described Gram‐positive flora. While positive cultures are reported in approximately 75% of cases, typically dominated by *Staphylococcus* species followed by Gram‐negative organisms such as *Pseudomonas* [10], only 63.5% of cultures in our cohort were positive. This discrepancy may be related to institutional practices, including prolonged perioperative antibiotic prophylaxis, which may also contribute to lower culture yield. In addition, the relatively high rate of resistant organisms observed in our cohort compared to prior reports [11] underscores the importance of tailoring empiric and targeted antibiotic therapy to local microbiologic profiles.

Beyond infection, we adopted a broader definition of “implants at risk,” including cases of skin necrosis, wound dehiscence, and implant exposure. These conditions are well‐recognized precursors to implant loss, even in the absence of overt infection [12, 13]. Prior studies suggest that early intervention in such cases may reduce device failure rates [14]. Traditionally, management of implant‐related complications has relied on explantation followed by delayed reconstruction. While effective in controlling infection, this approach carries significant reconstructive and patient‐related drawbacks, including soft tissue contraction, loss of the breast pocket, and lower rates of reconstruction completion [13]. Consistent with prior reports demonstrating that only up to 40% of patients ultimately undergo successful reconstruction following explantation [15], our findings show a markedly lower rate of final reconstruction in the explanted group. Similarly, large database analyses have demonstrated reduced reconstruction completion rates in patients with postoperative infection [2].

These findings support the growing body of evidence favoring salvage strategies to avoid explantation [4, 6, 16, 17]. Various techniques have been described, including antibiotic therapy, surgical debridement, implant exchange, irrigation systems, and negative pressure wound therapy. Continuous or intermittent implant pocket irrigation and adjunctive therapies such as negative pressure wound therapy have shown promising results in selected cases [18–22]. However, no standardized protocol has been established.

Our protocol emphasizes early, stepwise intervention while preserving the original implant whenever feasible. This approach differs from others by including a broader spectrum of implant‐threatening conditions and prioritizing implant retention without routine exchange. Despite the inclusion of more complex cases, including necrosis, dehiscence, and implant exposure, our salvage rates are comparable to those reported in the literature.

As most implant infections are traditionally attributed to Gram‐positive organisms, empiric treatment commonly includes cephalosporins and vancomycin [14, 23]. In our cohort, the predominance of Gram‐negative organisms prompted the addition of ciprofloxacin to standard regimens. However, increasing resistance among *Pseudomonas* species has been reported, particularly in the presence of tissue compromise such as flap necrosis, which may necessitate escalation to broader‐spectrum agents [15]. These findings further highlight the importance of adapting treatment strategies to evolving microbiologic trends.

This study has several limitations. Its retrospective design introduces potential selection bias, and the relatively small number of complication cases limits statistical power. Although the median follow‐up was 386 days, six patients (3.0%) had follow‐up shorter than 30 days. This may have resulted in incomplete ascertainment of final reconstructive outcomes in this small subset and should be considered when interpreting the findings.

Clinical decision‐making at the time of presentation was not standardized, although retrospective classification according to CDC criteria aimed to mitigate this variability. In addition, changes in surgical practice over time, including the transition from submuscular to prepectoral reconstruction, may have influenced complication rates.

Finally, long‐term outcomes such as capsular contracture were not systematically captured.

The potential trade‐off of salvage strategies, including the risk of late complications such as capsular contracture, should also be considered. Although capsular contracture was observed in a subset of patients, incomplete documentation and variable follow‐up preclude definitive conclusions.

In summary, our findings support a protocol‐based salvage approach for implant‐threatening complications, demonstrating that implant preservation is feasible and associated with improved rates of final reconstruction. Early, individualized management tailored to clinical presentation and microbiologic characteristics may optimize outcomes. Further prospective studies are needed to refine salvage criteria and establish standardized treatment algorithms.

## Funding

No funding was received for this manuscript.

## Conflicts of Interest

The authors declare no conflicts of interest.

## Data Availability

The data that support the findings of this study are available on request from the corresponding author. The data are not publicly available due to privacy or ethical restrictions.
